# A simplified prevention bundle with dual hand hygiene audit reduces early-onset ventilator-associated pneumonia in cardiovascular surgery units: An interrupted time-series analysis

**DOI:** 10.1371/journal.pone.0182252

**Published:** 2017-08-02

**Authors:** Kang-Cheng Su, Yu Ru Kou, Fang-Chi Lin, Chieh-Hung Wu, Jia-Yih Feng, Shiang-Fen Huang, Tao-Fen Shiung, Kwei-Chun Chung, Yu-Hsiu Tung, Kuang-Yao Yang, Shi-Chuan Chang

**Affiliations:** 1 Department of Chest Medicine, Taipei Veterans General Hospital, Taipei City, Taiwan, ROC; 2 Institute of Physiology, School of Medicine, National Yang-Ming University, Taipei City, Taiwan, ROC; 3 Center of Sleep Medicine, Taipei Veterans General Hospital, Taipei City, Taiwan, ROC; 4 Division of Infectious Disease, Department of Internal Medicine, Taipei Veterans General Hospital, Taipei City, Taiwan, ROC; 5 Nursing Department, Taipei Veterans General Hospital, Taipei City, Taiwan, ROC; 6 Genome Research Center, School of Medicine, National Yang-Ming University, Taipei City, Taiwan, ROC; 7 Institute of Emergency and Critical Care Medicine, School of Medicine, National Yang-Ming University, Taipei City, Taiwan, ROC; Azienda Ospedaliero Universitaria Careggi, ITALY

## Abstract

**Background:**

To investigate the effect of a simplified prevention bundle with alcohol-based, dual hand hygiene (HH) audit on the incidence of early-onset ventilation-associated pneumonia (VAP).

**Methods:**

This 3-year, quasi-experimental study with interrupted time-series analysis was conducted in two cardiovascular surgery intensive care units in a medical center. Unaware external HH audit (eHH) performed by non-unit-based observers was a routine task before and after bundle implementation. Based on the realistic ICU settings, we implemented a 3-component bundle, which included: a compulsory education program, a knowing internal HH audit (iHH) performed by unit-based observers, and a standardized oral care (OC) protocol with 0.1% chlorhexidine gluconate. The study periods comprised 4 phases: 12-month pre-implementation phase 1 (eHH+/education-/iHH-/OC-), 3-month run-in phase 2 (eHH+/education+/iHH+/OC+), 15-month implementation phase 3 (eHH+/education+/iHH+/OC+), and 6-month post-implementation phase 4 (eHH+/education-/iHH+/OC-).

**Results:**

A total of 2553 ventilator-days were observed. VAP incidences (events/1000 ventilator days) in phase 1–4 were 39.1, 40.5, 15.9, and 20.4, respectively. VAP was significantly reduced by 59% in phase 3 (*vs*. phase 1, incidence rate ratio [IRR] 0.41, *P* = 0.002), but rebounded in phase 4. Moreover, VAP incidence was inversely correlated to compliance of OC (r^2^ = 0.531, *P* = 0.001) and eHH (r^2^ = 0.878, *P* < 0.001), but not applied for iHH, despite iHH compliance was higher than eHH compliance during phase 2 to 4. Compared to eHH, iHH provided more efficient and faster improvements for standard HH practice. The minimal compliances required for significant VAP reduction were 85% and 75% for OC and eHH (both *P* < 0.05, IRR 0.28 and 0.42, respectively).

**Conclusions:**

This simplified prevention bundle effectively reduces early-onset VAP incidence. An unaware HH compliance correlates with VAP incidence. A knowing HH audit provides better improvement in HH practice. Accordingly, we suggest dual HH audit and consistent bundle performance does matter in quality-of-care VAP prevention.

## Introduction

Ventilator-associated pneumonia (VAP) is one of the most common nosocomial infection in the intensive care unit (ICU) [1]. It occurs at an estimated rate of 1% to 3% per day after initiation of mechanical ventilation (MV), and the cumulative incidence increases if MV period is prolonged [2]. Despite wide variation of VAP incidence (5 to 67%) [1], depending on the cases selected and the diagnostic criteria used, VAP is generally associated with more antibiotic consumption, greater hospital costs, longer MV duration and ICU stay, and, eventually, higher ICU and hospital mortality [1, 3].

The International Nosocomial Infection Control Consortium (INICC), founded by Dr. Victor D. Rosenthal in 1998, provides a famed international collaborative program to promote evidence-based infection control in hospitals with limited resource or insufficient experience in different area or countries [4]. Take Chinese population for example, the INICC surveillance data showed that VAP incidence was around 20.8 to 24.1 (events/1000 MV days) in different prospective cohorts [5, 6]. Recently, the INICC reported the VAP incidence, from a prospective, 6-year, multi- national study including 861,284 ICU patients, was higher than that in the last report from the Centers for Disease Control and Prevention National Healthcare Safety Network (CDC-NHSN) (13.1 *vs*. 0.9) [7]. The extensive INICC reports may provide a reference incidence for each ICU to head for better VAP prevention.

VAP prevention, regarded as infection control measures, has been identified as a safety issue for ICU patients. Several interventions have been proposed to reduce the incidence of VAP, including head-of-bed (HOB) elevation [8], daily sedation vacation [9], daily trial of ventilator weaning [9], alcohol hand hygiene (HH) [10], staff education program [11], and oral care (OC) with chlorhexidine gluconate (CHG) [12, 13]. VAP bundle, a combined approach with some selective interventions, has been shown to reduce VAP incidence effectively [14]. The incidence of VAP has become a quality indicator in many healthcare systems. However, the bundle components may vary according to available resources, facilities, and patients’ characteristics in different settings. Therefore, each ICU should establish its own practical “VAP bundle” as a central commitment to improve patient safety. Moreover, each bundle component must be individually validated for minimal compliance required for effective bundle performance.

Since VAP occurrence after cardiac surgery links to poor prognosis [15], VAP prevention has been part of routine care in our cardiovascular surgery intensive care units (CVSUs). The most common measures include OC and HH. However, in our CVSUs there existed some shortages, including lack of VAP education, no standardized procedures and compliance audit for OC, low HH compliance (around 71%) audited by institutional infection control center, and no well-estimated VAP incidence. Therefore, monitoring VAP incidence and systematic implementation of VAP bundle is critical for improving quality of care. Instead of a complex bundle beyond our capability, we hypothesized that establishing a simplified 3-component VAP bundle based on our own ICU settings with strict audit of bundle performance could reduce VAP incidence. In addition, we also investigated the effect of bundle compliance on VAP incidence and ICU outcomes.

## Material and methods

### Study design and settings

This quasi-experimental study with interrupted time-series (ITS) analysis was conducted in two CVSUs (total 16 beds) in the tertiary hospital, Taipei Veterans General Hospital. The CVSUs managed more than 1000 admissions per year. In December 2012, we established a VAP prevention committee, which was composed of multidisciplinary healthcare workers (HCWs), including doctors of different specialties (cardiovascular surgeons, infection control specialists, pulmonologists, and intensivists), and nurses from the primary care team and institutional infection control center. The committee determined the bundle components, held the meeting every other month, reviewed periodic the bundle performance, and redressed execution problems during the study periods. The institutional review board of Taipei Veterans General Hospital approved the study (ID: 2013-10-002A) and waived requirement of patient informed consent because the entity of this study was a quality-improvement program.

### Patients and study outcomes

Patients admitted to CVSUs were potentially eligible if they were older than 20 years and mechanically ventilated with artificial airway (including endotracheal and tracheostomy tube). The patients were excluded if they met the following conditions: 1. pneumonia occurred before CVSU admission; 2. placed with an artificial airway and mechanically ventilated more than 2 days before CVSU admission; 3. stay in CVSUs less than 48 hours for any reason; 4. duration of MV less than 48 hours.

The primary objective was to reduce the incidence of early-onset VAP, defined as VAP occurrence within 7 days after commencement of MV. The secondary objectives included bundle compliance and ICU outcomes. The count of patient ventilator days ceased after 7 days have passed or if ventilator days were out of CVSU.

### VAP definition, diagnosis and classification

VAP diagnostic criteria, based on the US CDC-NHSN surveillance definition, were consistent throughout the study periods and were categorized into clinical or microbiological VAP (S1 Appendix) [16–18]. The diagnosis of VAP was initially evaluated independently by two pulmonologists from medical records in retrospective phase (phase 1), and by two primary treatment physicians during prospective phases (phase 2–4). In addition, the modified clinical pulmonary infection score (CPIS) [19], calculated by well-trained staff independently of the VAP evaluators, was applied to increase the diagnostic accuracy of VAP. The timing to calculate modified CPIS was the day on which patients were suspected to have VAP or patients without VAP but stopped counting ventilator days. All cases were finally confirmed by the expert team, which comprised senior pulmonologists with at least 5-year experience of full-time critical care. The initial 2 separate VAP evaluators might vary in different study months, but the members of expert team and CPIS rater were fixed throughout the whole study periods. VAP incidence was presented as VAP events per 1000 ventilator days.

### Prevention bundle and implementation protocol

**Fig 1:**
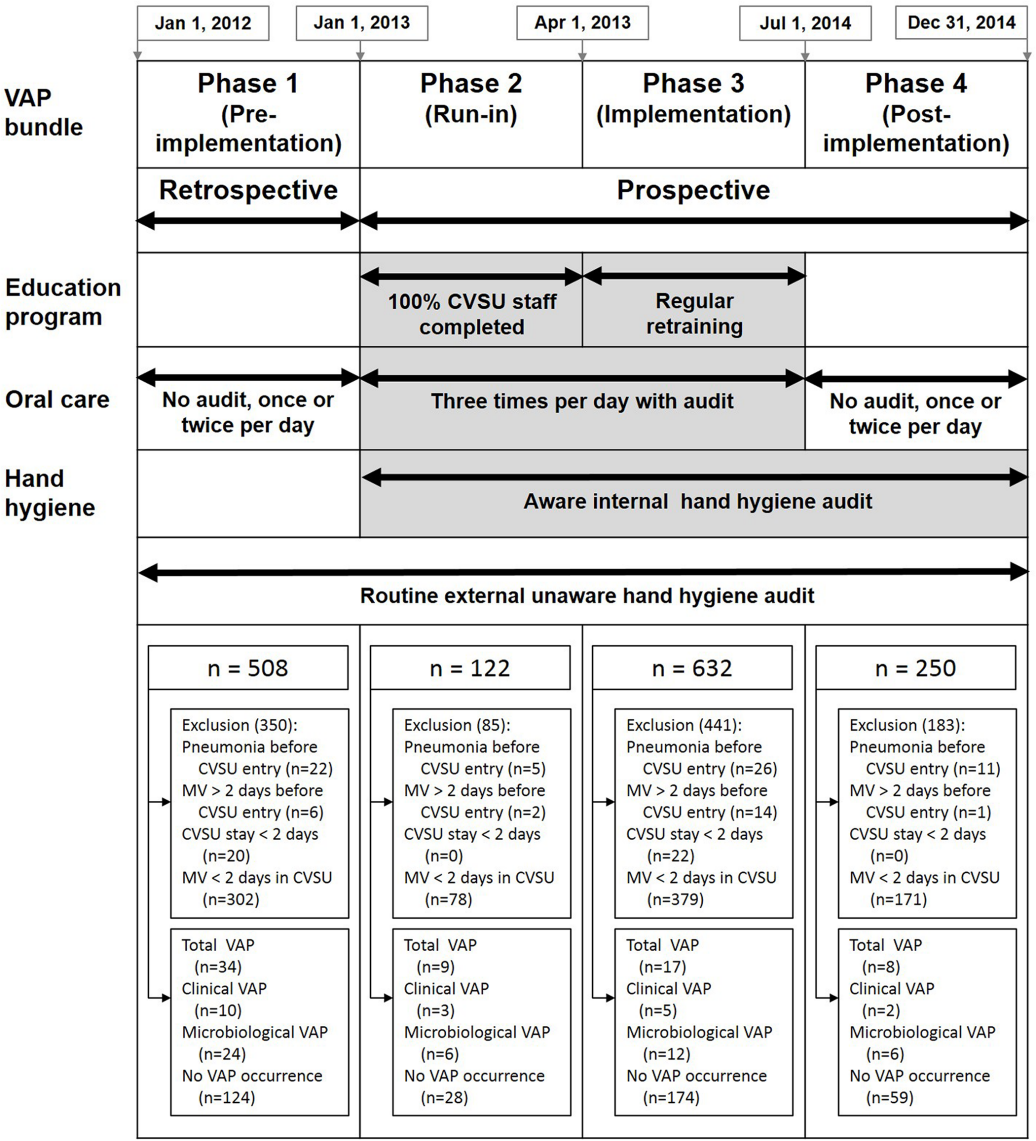
Study algorithm. Shadowed areas indicate periods of bundle implementation for each specific preventive measure. HCWs, healthcare workers; eHH, unaware external hand hygiene audited by non-unit-based observers; iHH, aware internal hand hygiene audited by unit-based observers.

An unaware external HH audit (eHH) performed by non-unit-based observers has been a routine and independent task before and after bundle implementation. Additionally, we implemented a 3-component bundle, which included: a compulsory education program (8-hour, lecture-based lessons for all HCWs in CVSU), a knowing internal HH audit (iHH) performed by unit-based observers, and a standardized oral care (OC) by 0.1% CHG toothbrushing and mouth washing [13, 20] with performance audited by checklists (S2 Appendix). The study periods comprised 4 phases—12-month pre-implementation phase 1 (eHH+/education-/iHH-/OC-), 3-month run-in phase 2 (eHH+/education+/iHH+/OC+), 15-month implementation phase 3 (eHH+/education+/iHH+/OC+), and 6-month post-implementation phase 4 (eHH+/education-/iHH+/OC-)—between 2012 and 2014 (Fig 1). During phase 1, VAP incidence was determined as baseline data by retrospective review of medical records. During phases 2 to 4, the study was conducted prospectively. Phase 2 was a preparation period to educate CVSU staffs being familiar with bundle components, thereafter, followed by systematic bundle implementation with strict compliance audit during phase 3. Finally, during phase 4, education and OC were withdrawn to observe the change of VAP incidence. The performance of HH and audit followed WHO guideline [21]. Bundle reminders were posted in the CVSU to engage HCWs’ attention regarding bundle care. Additionally, some other common preventive practices, such as HOB elevation, sedation vacation, daily cuff pressure control, etc. were available on the decision of the primary treatment team in accordance with the variable and individualized situations.

### Bundle compliance

**Fig 2:**
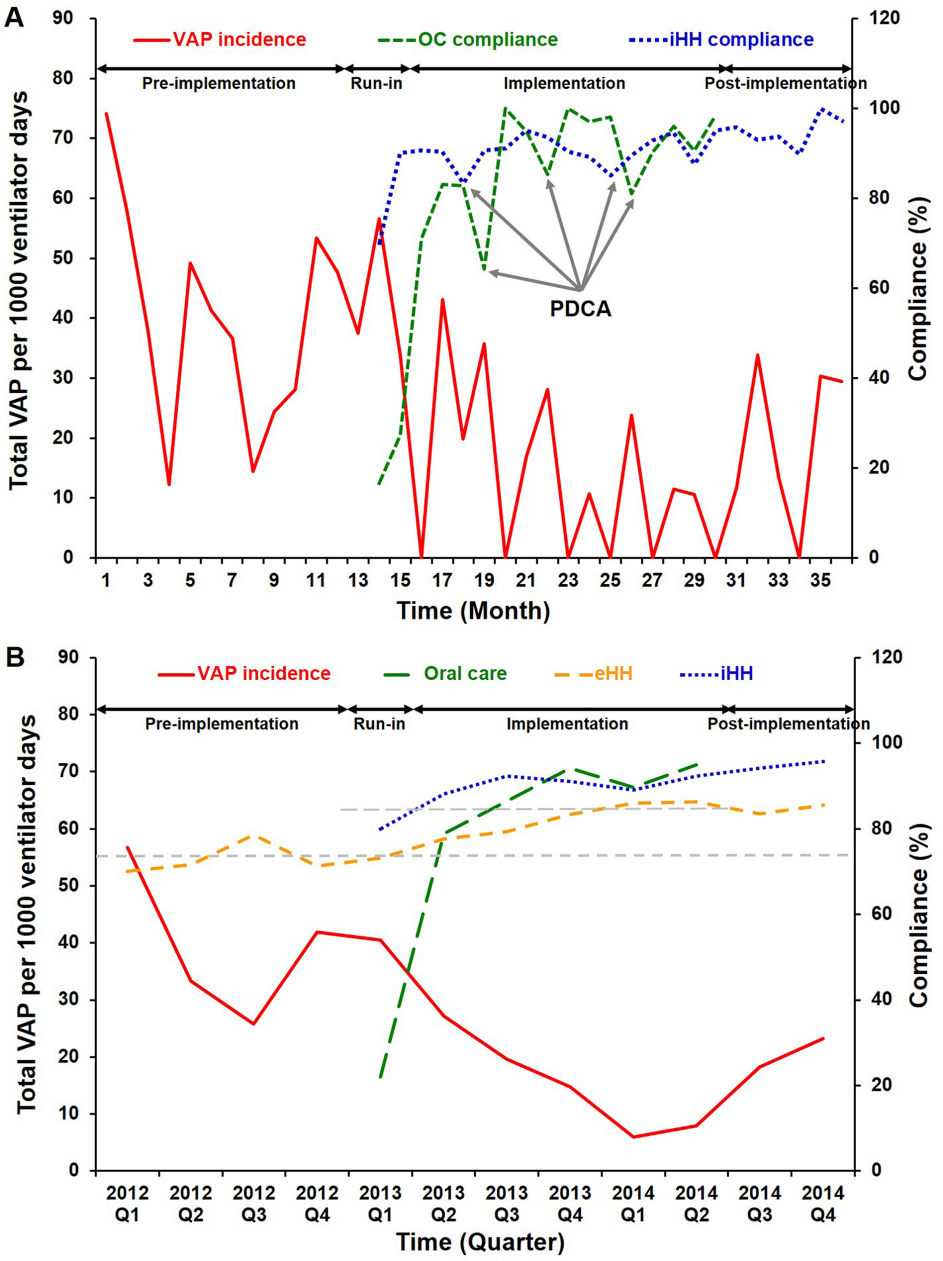
Incidence of total ventilator-associated pneumonia (VAP) during different study periods. Total VAP incidence (events/1000 ventilator days) and compliance of oral care (OC), aware internal hand hygiene audit (iHH) as well as unaware external hand hygiene audit (eHH) were illustrated at monthly intervals (A) and at quarterly intervals (B). PDCA means plan-do-check-act cycle in case of decrease in compliance monitoring.

The eHH compliance was monitored in turn by well-trained nursing observers of the institutional infection control center at unpredictable times and was announced quarterly. After bundle implementation, the compliance for iHH and OC was undertaken by unit-based-observers, usually well-experienced nursing leaders, who provided real-time feedback to ensure quality of care and announced compliance monthly (S2 Appendix). We applied Plan-Do-Check-Act (PDCA) cycles [22] to specify executive problems and maximize bundle compliance (Fig 2A).

### Statistical analyses

Statistics were analyzed using SPSS version 17.0 (SPSS, Inc., Chicago, IL). Data are presented as mean ° SD or 95% conference interval. ANOVA test or t-test was used to compare continuous variables between different groups, and Chi-square or Fisher-exact test to compare categorical data. Incidence was analyzed using Poisson regression. Changes of VAP incidence and bundle compliance were assessed using monthly or quarterly data throughout the study periods and the correlation between each other was analyzed using Spearman’s rank test. Poisson regression treated each single VAP incidence as an independent variable, which might ignore its underlying secular trend. For example, the VAP incidence might be interfered with external time-varying confounders or autocorrelation (eg. high incidence trends to follow high incidence and vice versa). ITS analysis takes the effect of secular trend into account. Autoregressive integrated moving average (ARIMA) model is one of the most common ITS analysis [23–26]. Therefore, ARIMA was applied to analyze the time evolution of the monthly VAP incidence [27]. All statistical tests were two-sided and evaluated at the 0.05 level of significance.

## Results

### Patient characteristics

A total of 1512 eligible patients were enrolled, accounting for 2553 ventilator days and 68 early-onset VAP events (Fig 1). The baseline characteristics among patients of different phases were similar (Table 1). The most common comorbidities were hypertension (78%), followed by coronary arterial disease (49%) and heart failure (38%), and 85% of patients were ventilated because of post-surgery care. The most common surgery was valvular repair (26%), followed by coronary artery bypass graft (25%) and repair of thoracic aortic aneurysm (20%). For total patients, the CPIS was significantly higher in patients with VAP than that in those without VAP (8.1 ± 1.9 *vs*. 3.9 ± 1.8, *P* < 0.001, S1 Fig).

**Table 1:** Demographic characteristics in different study phases.

Characteristics	Phase 1 (n = 158)	Phase 2 (n = 37)	Phase 3 (n = 191)	Phase 4 (n = 67)	*P* value
**Age, years**	64.8 ± 14.3	66.7 ± 14.9	66.3 ± 16.1	67.6 ± 16.6	0.606^a^
**Gender, M (%)**	114 (72.2)	21 (56.8)	125 (65.4)	46 (68.7)	0.271^b^
**APACHE II at ICU admission**	26.2 ± 6.1	26.5 ± 5.6	26.9 ± 5.2	27.4 ± 6.3	0.426^a^
**Comorbidity (%)**					
** Hypertension**	119 (75.3)	29 (78.4)	153(80.1)	52 (77.6)	0.864^b^
** Coronary arterial disease**	80 (50.6)	18 (48.6)	91 (47.6)	33 (49.3)	0.979^b^
** Heart failure**	59 (37.3)	16 (43.2)	69 (36.1)	28 (41.8)	0.487^b^
** Diabetes mellitus**	45 (28.5)	11 (29.7)	51 (26.7)	20 (29.9)	0.961^b^
** Chronic kidney disease**	30 (19.0)	6 (16.2)	30 (15.7)	9 (13.4)	0.721^b^
**Chronic lung disease**^**c**^	12 (7.6)	3 (8.1)	14 (7.3)	5 (7.5)	0.992^b^
**Reason for mechanical ventilation (%)**					
** Post elective surgery**	74 (46.8)	24 (64.9)	117 (61.3)	39 (59.1)	0.060^b^
** Post emergent surgery**	57 (36.1)	7 (18.9)	48 (25.1)	19 (28.8)	0.053^b^
** Cardiogenic shock**	11 (6.9)	3 (8.1)	11 (5.8)	4 (6.0)	0.933^b^
** Others**	16 (10.1)	3 (8.1)	15 (7.9)	5 (7.5)	0.941^b^
**Type of surgery (%)**					
**Valve repair**^**d**^	42 (26.6)	8 (22.9)	53 (27.7)	17 (25.3)	0.856^b^
** CABG**	44 (27.8)	6 (17.1)	52 (27.2)	11 (16.4)	0.068^b^
** TAA repair**	34 (22.8)	8 (21.6)	34 (17.8)	14 (20.9)	0.839^b^
** Endovascular surgery**	14 (8.9)	3 (8.1)	22 (11.5)	6 (8.9)	0.804^b^
** AAA repair**	8 (5.1)	2 (5.4)	8 (4.2)	3 (4.5)	0.984^b^
**Supporting device**^**e**^	12 (7.6)	5 (13.5)	16 (8.4)	6 (9.0)	0.439^b^

Continuous variables are presented as mean ± SD. Categorical variables are presented as counts and percentages in parentheses.

APACHE, acute physiology and chronic health evaluation; ICU, intensive care unit; CABG, coronary artery bypass graft; TAA, thoracic aortic aneurysm; AAA, abdominal aortic aneurysm.

^a^ANOVA test.

^b^Chi-square test.

^c^Chronic lung disease includes chronic pulmonary obstructive disease, asthma, bronchiectasis, and interstitial lung disease.

^d^Valve repair indicates valvular surgery for mitral and aortic valve.

^e^Supporting device includes extracorporeal membrane oxygenation and ventricular assisted device.

### VAP incidence and ICU outcomes

**Fig 3:**
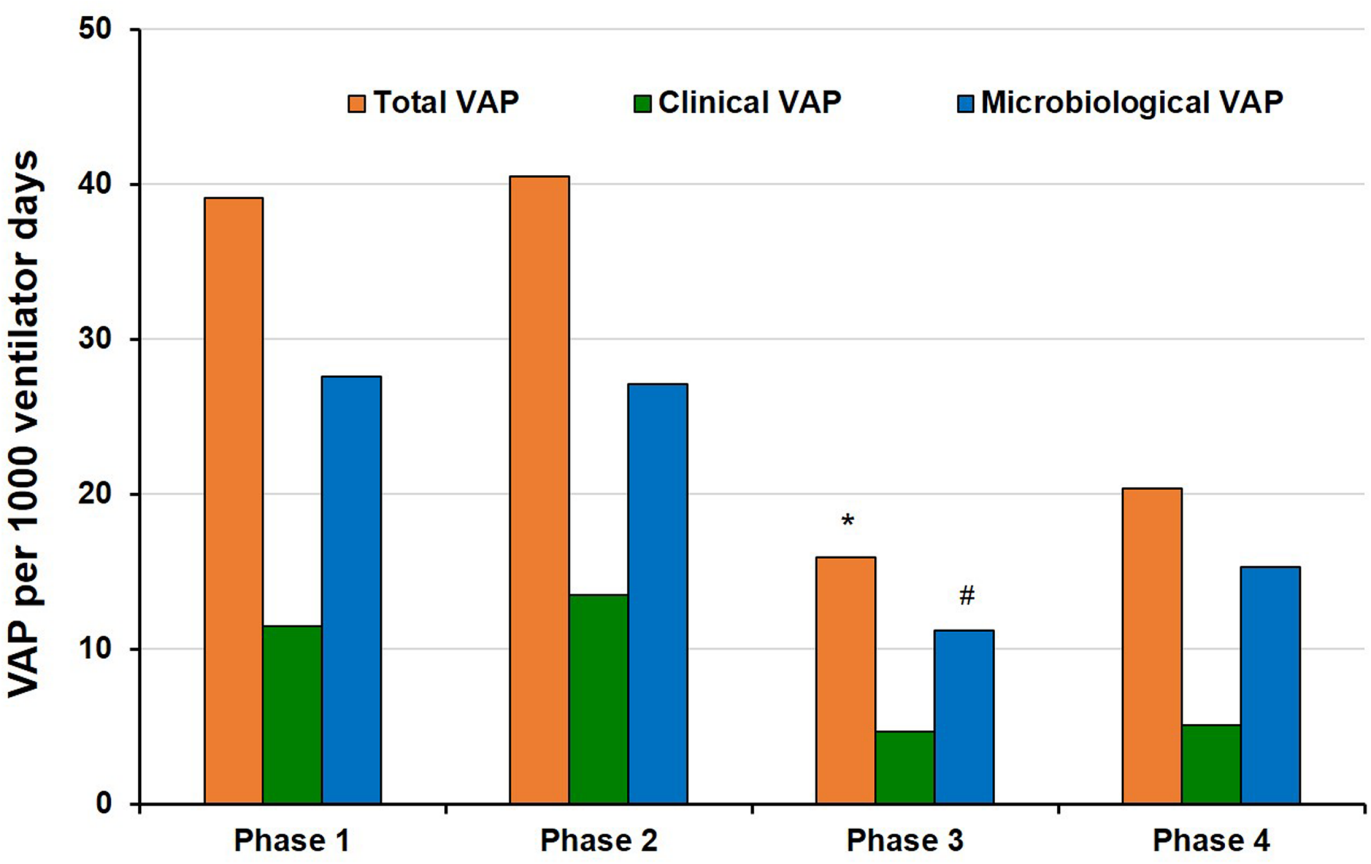
The total, clinical and microbiological VAP incidences during different study periods. Poisson regression, * *P* < 0.05, *vs*. phases 1 and 2, respectively; ^#^ *P* < 0.05, *vs*. phase 1.

Systematic bundle implementation resulted in steady and sustained decrease in total VAP incidence (Fig 2**)**. Total and microbiological VAP incidences in phase 1 to 4 were 39.1 and 27.6, 40.5 and 27.1, 15.9 and 11.2, 20.4 and 15.3, respectively (Fig 3). The compliance of OC and iHH during phase 3 significantly improved (*vs*. phase 2, Table 2), and there was significant reduction of incidence for total VAP during phase 3 (*vs*. phases 1 & 2, *P* = 0.002 & 0.033, respectively) and for microbiological VAP (*vs*. phase 1, *P* = 0.008) (Fig 3). The total VAP incidence during phase 3 reached a 59% reduction in comparison with that during phase 1 (incidence rate ratio [IRR] 0.41, *P* = 0.002, 95% conference interval 0.23–0.73), which accounted for a number needed to treat (NNT) of 8. However, during phase 4, the total VAP incidence tended to rebound and failed to sustain significant improvement (*vs*. phase 1, *P* = 0.078, Figs 2 and 3), such as that during phase 3. For ITS analysis, the ARIMA (1,0,1) served as the best fit model, in which the R^2^ was 0.501, and the Ljung-Box Q statistic indicated the absence of statistically significant autocorrelations in residuals (Q = 15.535, df = 16, significance = 0.486). That was, the VAP incidence presented as a stationary time series data. The monthly VAP incidence was independent, not been interfered with autocorrelation. The VAP bundle had an effect significantly greater than the underlying secular trend. The results of Poisson regression were comparable with those of ITS analysis. The detailed information was shown in S3 Appendix.

**Table 2:** ICU outcomes in different study phases.

	Phase 1 (n = 158)	Phase 2 (n = 37)	Phase 3 (n = 191)	Phase 4 (n = 67)	*P* value
**Outcomes in the first week of MV**					
** Total ventilator days**	5.5 ± 1.7	6.0 ± 1.3	5.6 ± 1.7	5.9 ± 1.6	0.248^a^
** ICU mortality (%)**	12 (7.6)	0	8 (5.3)	4 (6.0)	0.351^b^
** Successful extubation (%)**	73 (33.3)	14 (37.8)	88 (46.1)	23 (34.3)	0.055^b^
**Outcomes throughout ICU stay**					
** Total ventilator days**	12.2 ± 15.9	14.1 ± 15.1	12.4 ± 17.4	14.8 ± 19.9	0.702^a^
** ICU mortality (%)**	36 (22.8)	4 (10.8)	40 (20.9)	11 (16.4)	0.341^b^
** Successful extubation (%)**	120 (75.9)	29 (78.4)	146 (76.4)	54 (80.6)	0.882^b^
** Length of ICU stay, days**	13.7 ± 15.5	18.9 ± 15.8	14.6 ± 17.9	17.5 ± 20.4	0.228^a^
**Total antibiotic days**^**c**^	10.0 ± 4.7	11.9 ± 3.5	10.6 ± 4.4	11.0 ± 4.3	0.092^a^
**Length of hospital stay, days**	37.4 ± 28.9	47.4 ± 30.4	41.8 ± 37.1	41.5 ± 30.7	0.331^a^
**Compliance, %**					
** Oral care**	-	22.0	88.8	-	< 0.001^b^
** Hand hygiene- external audit**	72.9	73.2	82.5	84.5	0.084^b^
** Hand hygiene- internal audit**	-	78.5	90.6	94.9	0.002^b^

Continuous variables are presented as mean ± SD. Categorical variables are presented as counts and percentages in parentheses.

ICU, intensive care unit; MV, mechanical ventilation.

^a^ANOVA test.

^b^Chi-square test.

^c^Total antibiotic days mean the total days of the patients receiving antibiotic treatment in the first 14 days after commencement of mechanical ventilation.

Regarding ICU outcomes, the total ventilator days, ICU stay, ICU mortality, and successful extubation rate showed no difference either in the first week of MV initiation or throughout the ICU course. The total antibiotic days in the first 14 days after commencement of MV were also similar (Table 2). The microbiological culture of endotracheal aspirates collected within 7 days of MV yielded gram-negative bacilli (36.6%), fungi (6.0%), and gram-positive cocci (2.9%), of which 9.1% were multi-drug-resistant strains. The number of negative cultures yield tended to be increased after bundle implementation (27.2%, 32.4%, 36.6%, 40.3% during phases 1, 2, 3, and 4, respectively) (S1 Table).

### Bundle compliance

**Fig 4:**
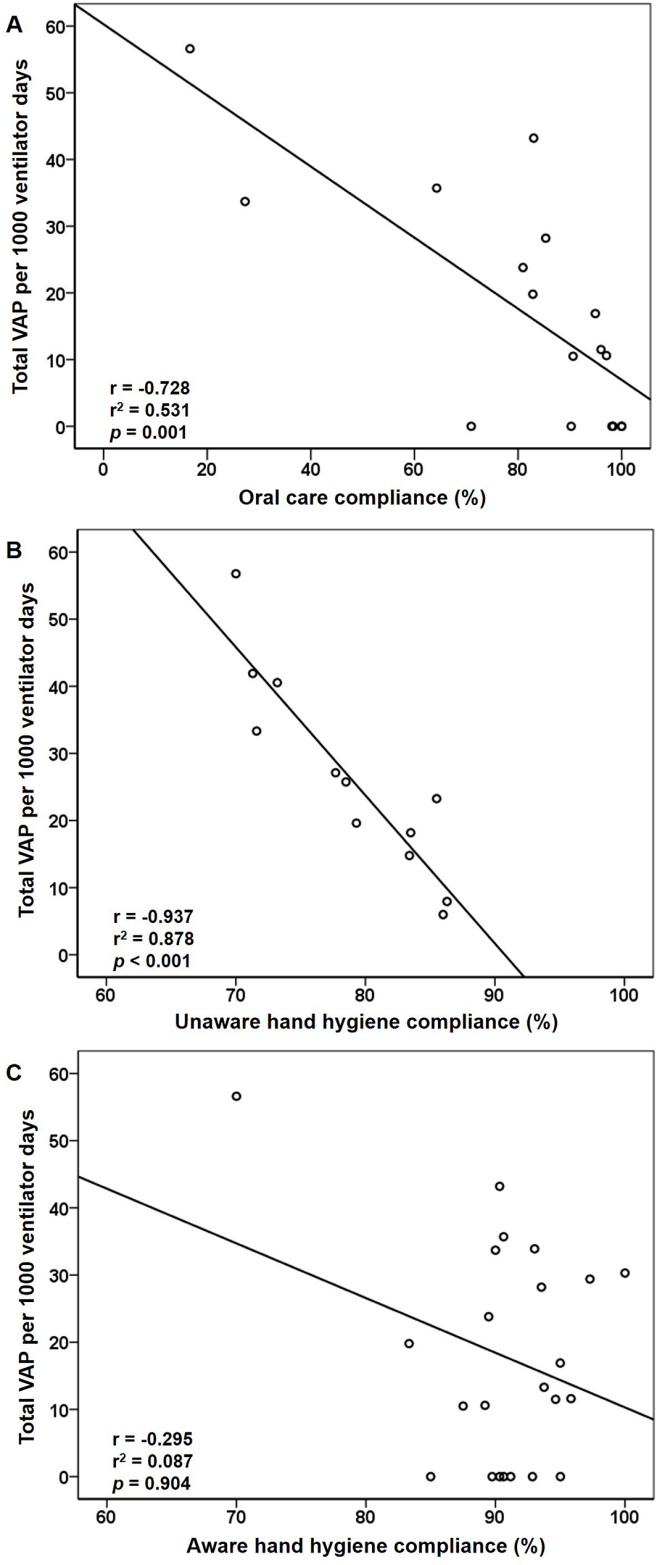
**Correlation between total VAP incidence and compliance for A, oral care; B, unaware external hand hygiene audit (eHH); and C, aware internal hand hygiene audit (iHH)** (Spearman’s correlation).

One-hundred percent of HCWs completed at least one 3-hour education lessons during phase 2, and around 90% of HCWs received a total of 5 hours of regular retraining sessions during phase 3. The compliance of OC and iHH was rapidly increased beyond the pre-set level of 80% at the end of phase 2, and maintained at a high level by means of PDCA cycles till the end of phase 3 **(**Fig 2A). Total VAP incidence was inversely correlated with the compliances for OC (r^2^ = 0.531, *P* = 0.001, Fig 4A) and eHH (r^2^ = 0.878, *P* < 0.001, Fig 4B), but not for iHH (r^2^ = 0.087, *P* = 0.904, Fig 4C). Compared to eHH, iHH provided a more efficient and faster improvement for standard HH practice (Fig 2B). Among HCWs of different specialties, doctors, nurses, and respiratory therapists showed significant improvement in eHH compliance (S2 Fig).

Furthermore, the minimal required compliances for different bundle components were determined. For OC, a compliance of 85% achieved a significant VAP reduction by 72% (IRR = 0.28, *P* = 0.004), accounting for an NNT of 7. Similar results were reproduced in eHH, but not in iHH. A minimal required eHH compliance of 75% resulted in 58% reduction and an NNT of 7 (IRR = 0.42, *P* < 0.001). The higher the bundle compliance, the more VAP events were prevented (Table 3).

**Table 3:** Total ventilator-associated pneumonia incidence stratified by variable cut-off value for different bundle component.

Compliance with variable cut-off value	Total VAP incidence^a^		Rate ratio (95% CI) (High *vs*. Low)	*P* value^b^
	≥ CO	< CO		
**Oral care**				
** 75%**	15.6	31.6	0.50 (0.21–1.16)	0.124
** 80%**	15.6	31.6	0.50 (0.21–1.16)	0.124
** 85%**	8.9	31.8	0.28 (0.11–0.71)	0.004
** 90%**	6.6	31.4	0.21 (0.07–0.62)	0.001
** 95%**	5.3	25.2	0.21 (0.05–0.89)	0.009
**Hand hygiene- external audit**				
** 75%**	18.3	43.1	0.42 (0.26–0.68)	< 0.001
** 80%**	13.8	35.1	0.39 (0.22–0.71)	0.001
** 85%**	11.8	31.1	0.38 (0.17–0.83)	0.006
** 90%**	0	26.6	-	-
**Hand hygiene- internal audit**				
** 75%**	18.1	56.6	0.32 (0.10–1.05)	0.105
** 80%**	18.1	56.6	0.32 (0.10–1.06)	0.105
** 85%**	18.6	24.3	0.76 (0.29–2.01)	0.597
** 90%**	19.3	19.5	0.99 (0.47–2.04)	0.976
** 95%**	17.3	19.8	0.88 (0.34–2.27)	0.781

CO, cut off value; CI, confidence interval.

^a^VAP incidence indicates VAP events per 1000 ventilator days.

^b^Poisson regression.

## Discussion

This simplified 3-component bundle successfully reduced the incidence of early-onset VAP in CVSUs. The major findings included minimal compliances required for individual bundle component, external audit better than internal audit for HH, and inconsistent bundle performance resulting in rebounding of VAP incidence. Establishment of a bundle care suited to its realistic settings does matter in quality-of-care clinical practice.

Efforts to improve HH are the leading measures to prevent nosocomial infection. The WHO proposes that direct observation and feedback regarding HH by a trained observer is the gold standard for establishing compliance rates [21], which usually contributes to small but potentially pivotal improvements of infection control in professional practice [28]. With bundle implementation, both iHH and eHH compliance significantly improved. The difference between iHH and eHH was that the HCWs knew they were being observed (iHH) or not (eHH). Interestingly, the compliance of iHH was higher than that of eHH throughout the study (Fig 2B). This finding was comparable with those of recent reports, in all of which HH compliance was inflated during auditing by aware observers [29–32]. This phenomenon, the so-called Hawthorne effect, is often labeled as observer bias. This effect potentially explains why iHH compliance failed to correlate with VAP incidence in our results. Instead, eHH significantly correlated with VAP incidence, which indicated eHH was independent from observer bias and reliable to predict VAP control. Even so, aware observation (iHH) is also important because it provides an opportunity to establish real-time feedback and a more efficient HH practice, and may contribute to sustained improvement of HH [33]. Thus, dual HH audit was more proposed. In addition, each ICU should build up its own standardized HH protocol, such as the INICC HH program- including administrative support, supplies availability, education and training, reminders in the workplace, process surveillance, and performance feedback [4, 34] to ensure an effective and practical HH protocol based on its own resources.

One of the major pathogeneses of VAP is thought to be that microbe-laden secretions spread from upper airways downward into the lungs via microaspiration or endotracheal route [35, 36]. VAP pathogens were often genetically indistinguishable from strains isolated from the oral cavity [37], which indicates the importance of OC in VAP prevention. A recent large-scale meta-analysis concluded effective OC with CHG was associated with a 40% reduction of VAP development, accounting an NNT of 15 in critically ill adults [38]. Similarly, we reported that an OC compliance of 85% reduced VAP occurrence by 72%, with an NNT of 7. It may be questioned why unaware OC compliance was not monitored. It is technically difficult to accomplish because without close watching, we were not able to check all the required OC procedures. With close observation and real-time feedback by direct audit, we ensured high-quality OC and documented higher OC compliance was associated with lower VAP incidence. This result was similar to the recent study that 4-day protocolized OC was significantly superior to routine OC in VAP reduction [39]. Moreover, we observed that the negative microbiological culture rate of endotracheal aspirates gradually increased from phase 1 to 4. This phenomenon may reflect the changes of environment-microorganism relationships after implementation of HH and OC. The impact of such disinfection procedures on the microbiology of VAP deserves further investigation.

Previous studies highlighted the impact of bundle compliance on VAP reduction [40, 41], particularly aiming at the target compliance >95% for all components [22, 40]. Such a goal is very difficult to achieve, and some studies reported that even compliance lower than the target also achieved significant VAP reduction [17, 42], which indicates that there are some unmeasured factors executing positive impact on VAP reduction and the target compliance might be overestimated. It is reasonable to speculate different bundle components may have different impacts on VAP. Take OC, for example; our data showed a compliance of 90% reached its maximal effectiveness. For OC and eHH, minimal required compliances of 85% and 75%, respectively, were able to reach a significant VAP reduction. Universally targeting super-high compliance for each bundle component indeed increases unnecessary workload. Every bundle implementation should validate its own bundle effectiveness and build up its own optimal compliance target.

Most VAP studies reported successful VAP implementation, however, there was seldom study focusing on what would happen if VAP bundle was discontinued or performed inconsistently. We documented that withdrawing an education program and a strict OC performance resulted in loss of effective VAP reduction within 6 months, despite HH compliance remaining high. Similar results were reported, that VAP incidence was reduced with implementation of HH and daily CHG baths, but significantly rebounded again after withdrawal of daily CHG baths, in a 6-month post-implementation period [43]. Inconsistent bundle performance may flunk in maintaining low VAP incidence as early as 6 months. In the past years, the INICC has proposed a well-established multidimensional approach for VAP reduction and helped plenty of ICUs in Latin America, Europe, Eastern Mediterranean, Southeast Asia, and Western Pacific to effectively reduce VAP incidence by 31% to 79% [6, 27, 44–50]. The principle of the INICC program, including a bundle of infection-control interventions, education, outcome surveillance, process surveillance, feedback on VAP rates and consequences, performance feedback of process surveillance [4], can serve as a good model for effective bundle implementation and sustained VAP reduction.

We recognize that identifying VAP cases may be at a potential risk of bias because the interventions were not blinded and the clinical criteria of pneumonia are subjective. However, masking group-level interventions in quasi-experimental study is challenging, particularly in quality-improvement program [51]. Until new objective and universally accepted diagnostic tools are discovered, we have to acknowledge that no surveillance method can accurately predict the presence of histological pneumonia. In this study, dedicated efforts were made to minimize this potential bias by rigorous establishment of VAP diagnosis, which included the consistent VAP surveillance definition, comparably high CPIS (mean CPIS was 8.1) in VAP cases calculated by independent rater, and consensus strategy for VAP diagnosis (initially evaluated by 2 separate physicians, followed by confirmation of the fixed senior expert team). As to bundle components, daily sedation vacation, daily measurement of tracheal cuff pressure, HOB elevation, OC and eHH had been performed as routine tasks before bundle interventions. The compliance of the former two reached greater than 80%, but only around 71% for eHH. Surprisingly, we noted that the baseline VAP incidence in our CVSUs was relatively higher than that in previous reports. The causes might be difficult to elucidate and often multifactorial. Thus we speculated some of these reasons potentially capable of being improved, which comprised poor cognition for VAP prevention, high staff turnover (about 20 to 30% of staff were training interns, residents, nurses, and respiratory therapists), lack of standardized protocol and effective compliance for bundle prevention. Thus, we adopted education, OC, and dual-HH in consequence of no financial support and based on our realistic CVSU settings. Regarding head-of-bed elevation, which became problematic to execute in CVSUs because patients frequently experienced hemodynamic instability with or without support by extracorporeal membrane oxygenation and/or continuous renal replacement therapy support. Moreover, HOB elevation have been criticized for its effectiveness recently [52–54]. Thus, HOB elevation was left as a routine care without compliance audit. The more bundle components that were applied, the less adequate performance and sufficient compliance could be achieved [41]. Again, the prevention strategy should establish a reasonable goal and choose their own bundle components suitable for individual settings and available resources.

This study has limitations. First, our prevention bundle, similar to previous reports [15, 38, 55], did not contribute to significant improvement in ICU outcomes. The probable reasons include limited statistical power of small-sized study and the effectiveness of the bundle components we had selected. Second, our VAP bundle was implemented in a specific CVSU with high baseline VAP incidence, and was focused on 7-day VAP occurrence. The extrapolation of this simplified strategy to other ICU settings needs further validation. Finally, we demonstrated the association between VAP incidence and bundle components, not a causal link. The significant VAP reduction may in part be ascribable to a concurrent quality-of-care improvement by multidisciplinary teamwork.

## Conclusions

Instead of complicated bundle components or costly interventions, we present that a simplified strategy based on the realistic settings effectively reduced early-onset VAP incidence in CVSUs. The minimally required compliance of each proposed bundle component for VAP reduction should be validated individually to avoid excessive workload. Unaware HH compliance correlated with VAP incidence. Aware HH compliance might be inflated but it provided better improvement in HH practice. We suggest dual HH audit with consistent bundle performance may sustain good-practice at both the HCW and organization levels to prevent VAP occurrence.

## Supporting information

S1 Appendix VAP definition and classification. (DOCX)

S2 Appendix Detailed information regarding to education program, hand hygiene and compliance audit as well as standardized oral care. (DOCX)

S3 Appendix ARIMA analysis for monthly total VAP incidence. (DOCX)

S1 Table Microbiological spectrum in tracheal aspirates during different study phases. (DOCX)

S1 Fig The modified clinical pulmonary infection score categorized by the presence or absence of ventilator-associated pneumonia in total patients or in patients of different study phases. (DOCX)

S2 Fig Changes of compliance for unaware external hand hygiene audit (eHH) in different healthcare workers during different phases. (DOCX)

## Abbreviations

CHG: chlorhexidine gluconate
CVSU: cardiovascular surgery intensive care unit
eHH: unaware external hand hygiene audit
HCW: healthcare worker
HH: hand hygiene
HOB: head-of-bed
ICU: intensive care unit
iHH: aware internal hand hygiene audit
ITS: interrupted time-series Analysis
MV: mechanical ventilation
OC: oral care
VAP: ventilator-associated pneumonia.
