# Supplementary material for: A simplified prevention bundle with dual hand hygiene audit reduces early-onset ventilator-associated pneumonia in cardiovascular surgery units: An interrupted time-series analysis
Source: PLoS One. 2017 Aug 2;12(8):e0182252. doi: 10.1371/journal.pone.0182252 (PMC5540591; doi:10.1371/journal.pone.0182252)
Supplement: S1 Appendix — (DOCX) [file pone.0182252.s001.docx]

**S1 Appendix**

**VAP definition and classification**

VAP was defined as a pneumonia where the patient was on mechanical ventilation for more than two calendar days and the ventilator was in place on the date of event or the day before [1]. The definition of pneumonia was according to the Centers for Disease Control and Prevention/National Healthcare Safety Network (CDC/NHSN) surveillance definition [1, 2]. Pneumonia was identified by using a combination of radiologic, clinical and laboratory criteria. VAP was categorized into clinical or microbiological VAP according to criteria listed in Table A [1-3].

| **Table A. Criteria for diagnosis of ventilator-associated pneumonia.** | | | |
| --- | --- | --- | --- |
|  | **Radiology** | **Clinical/Symptoms/Laboratory** | **Microbiological evidence** |
| **Clinical VAP** | Two or more serial chest radiographs with at least one of the following:  • New or progressive and persistent infiltrate  • Consolidation  • Cavitation  NOTE: In patients without underlying pulmonary or cardiac disease (e.g., respiratory distress syndrome, broncho- pulmonary dysplasia, pulmonary edema, or chronic obstructive pulmonary disease), one definitive chest radiograph is acceptable. | At least one of the following:  • Fever (>38 °C or >100.4 °F)  • Leukopenia (<4000 WBC/mm^3^) or leukocytosis (≧12,000 WBC/mm^3^)  • For adults ≥70 years old, altered mental status with no other recognizable cause  and  at least two of the following:  • New onset of purulent sputum, or change in character of sputum, or increased respiratory secretions, or increased suctioning requirements  • New onset or worsening cough, or dyspnea, or tachypnea  • Rales or bronchial breath sounds  • Worsening gas exchange (e.g., O_2_ desaturation [e.g., PaO_2_/FiO_2_ ≦240], increased oxygen requirements, or increased ventilator demand) | Not necessary |
| **Microbiological VAP** | Same as above criteria | At least one of the following:  • Fever (>38 °C or >100.4 °F)  • Leukopenia (<4000 WBC/mm^3^) or leukocytosis (≧12,000 WBC/mm^3^)  • For adults ≥70 years old, altered mental status with no other recognizable cause  and  at least one of the following:  • New onset of purulent sputum, or change in character of sputum, or increased respiratory secretions, or increased suctioning requirements  • New onset or worsening cough, or dyspnea, or tachypnea  • Rales or bronchial breath sounds  • Worsening gas exchange (e.g., O_2_ desaturation [e.g., PaO_2_/FiO_2_ ≦240], increased oxygen requirements, or increased ventilator demand) | At least **one** of the following:  • Positive growth in blood culture not related to another source of infection  • Positive growth in culture of pleural fluid  • Positive quantitative culture from minimally-contaminated LRT specimen  (e.g., BAL or protected specimen brushing)  • ≥5% BAL-obtained cells containing intracellular bacteria on direct microscopic exam (e.g., Gram’s stain)  • Histopathologic exam showing at least one of the following evidences of pneumonia:  − Abscess formation or foci of consolidation with intense PMN accumulation in bronchioles and alveoli  − Positive quantitative culture of lung parenchyma  − Evidence of lung parenchyma invasion by fungal hyphae or pseudohyphae |
| LRT, low respiratory tract; BAL, bronchoalveolar lavage; PMN, polymorphonuclear leukocytes.  Definition of ventilation-associated pneumonia (VAP) means in persons who had a device to assist or control respiration continuously through a tracheostomy or by endotracheal intubation within the 48-hour period before the onset of infection [2].  Criteria for clinical and microbiological VAP is based on the CDC/NHSN surveillance definition [2]. | | | |

**Reference**

1. April 2013 CDC/NHSN Protocol Corrections, Clarification, and Additions. 2013. Available:http://www.cdc.gov/nhsn/pdfs/pscmanual/9pscssicurrent.pdf.

2. Horan TC, Andrus M, Dudeck MA. CDC/NHSN surveillance definition of health care-associated infection and criteria for specific types of infections in the acute care setting. Am J Infect Control. 2008;36(5):309-32. doi: S0196-6553(08)00167-3 [pii]

10.1016/j.ajic.2008.03.002 PMID: 18538699.

3. Morris AC, Hay AW, Swann DG, Everingham K, McCulloch C, McNulty J, et al. Reducing ventilator-associated pneumonia in intensive care: impact of implementing a care bundle. Crit Care Med. 2011;39(10):2218-24. doi: 10.1097/CCM.0b013e3182227d52 PMID: 21666444.
