# Supplementary material for: A simplified prevention bundle with dual hand hygiene audit reduces early-onset ventilator-associated pneumonia in cardiovascular surgery units: An interrupted time-series analysis
Source: PLoS One. 2017 Aug 2;12(8):e0182252. doi: 10.1371/journal.pone.0182252 (PMC5540591; doi:10.1371/journal.pone.0182252)
Supplement: S2 Appendix — (DOCX) [file pone.0182252.s002.docx]

**S2 Appendix**

**Compulsory education program**

The major contents of this lecture-based, compulsory educational program comprised overview of ventilator-associated pneumonia (VAP), including topics such as incidence, impact of VAP in the intensive care unit (ICU), the importance of VAP prevention, evidence-based VAP prevention components and maintenance of high bundle compliance. We focused on evidence-based bundle components and compliance monitoring. The timing and procedures for hand hygiene (HH) [1] and oral care (OC) with 0.1% chlorhexidine gluconate (CHG) [2, 3] were standardized and introduced to all healthcare workers (HCWs, including doctors, nurse, respiratory therapists, and all of the others worked in the ICU) by video demonstration and bed-side practice. Bundle reminders, such as small stickers and large posters, were posted at bedside and on the ICU door and wall to engage HCWs’ attention for bundle implementation. All the HCWs required a total of at least 3-hour lessons during phase 2 and 5-hour regular retraining sessions during phase 3. The goal of the education program was for HCWs to recognize and became familiar with bundle components in phase 2, and moreover, to provide HCWs with feedback in the form of the preliminary results of bundle implementation and reinforce motivation for bundle prevention in phase 3.

**Hand hygiene and compliance audit**

The performance of HH and audit followed WHO guideline [1]. HH was performed using alcohol-based (70% isopropyl alcohol) hand rubbing or hand washing with CHG soap solution (40 mg/ml) when visibly soiled [4]. Unaware HH compliance was audited by well-trained nursing staff from the hospital-affiliated infection control center, who in turn performed unscheduled ward rounds and made covert observations at unpredictable times. In contrast, aware HH compliance was audited by the ICU-affiliated senior or leading nurses, who made overt observations at randomized times. Only all the procedures met the standards of the WHO five moments for HH by checklist records was considered compliant. The compliance was announced monthly for iHH and quarterly for eHH during study periods.

**Standardized oral care**

OC was performed by gently brushing patients’ teeth, gingiva, buccal mucosa and tongue, using suction toothbrush with 30 ml of 0.1% CHG for 30 seconds, followed by oral irrigation with 10 ml of 0.1% CHG for 10 seconds, repeating 3 times. OC was done every 8 hours each day while the patient was placed in head-up position (> 30 degrees, unless contraindicated), with a plastic, soft, dental suction tube hung on the mouth for continuous suction to avoid aspiration. OC was contraindicated in case of swelling, ulcers or bleeding at the buccal mucosa, tongue, and gingiva. All nursing staff were required to be trained in and familiar with standardized OC. The compliance of OC was audited by checklist using the following table (Table B).

| **Table B. Checklist for oral care.** | | | | | | | | | | | | | | | | | | | | | |
| --- | --- | --- | --- | --- | --- | --- | --- | --- | --- | --- | --- | --- | --- | --- | --- | --- | --- | --- | --- | --- | --- |
| **Admission Date___________** | **Day 1** | | | **Day 2** | | | **Day 3** | | | **Day 4** | | | **Day 5** | | | **Day 6** | | | **Day 7** | | |
| Item Period | I | II | III | I | II | III | I | II | III | I | II | III | I | II | III | I | II | III | I | II | III |
| 1. Performance of oral care every 8 hours |  |  |  |  |  |  |  |  |  |  |  |  |  |  |  |  |  |  |  |  |  |
| 1. Gently brushing with 30 ml of 0.1% CHG for 30 s |  |  |  |  |  |  |  |  |  |  |  |  |  |  |  |  |  |  |  |  |  |
| 1. Oral irrigation with 10 ml of 0.1% CHG for 10 s |  |  |  |  |  |  |  |  |  |  |  |  |  |  |  |  |  |  |  |  |  |
| 4. Repeat steps 2 & 3 three times |  |  |  |  |  |  |  |  |  |  |  |  |  |  |  |  |  |  |  |  |  |
| Remarks |  |  |  |  |  |  |  |  |  |  |  |  |  |  |  |  |  |  |  |  |  |
| Period I: 8AM-4PM. Period II: 4PM-12AM. Period III: 12AM-8AM. *CHG* chlorhexidine gluconate | | | | | | | | | | | | | | | | | | | | | |

**References**

1. World Health Organization. WHO guidelines on hand hygiene in health care. 2009. Available:http://whqlibdoc.who.int/publications/2009/9789241597906_eng.pdf?ua=1.

2. Munro CL, Grap MJ, Jones DJ, McClish DK, Sessler CN. Chlorhexidine, toothbrushing, and preventing ventilator-associated pneumonia in critically ill adults. Am J Crit Care. 2009;18(5):428-37; quiz 38. doi: 10.4037/ajcc2009792 PMID: 19723863.

3. Houston S, Hougland P, Anderson JJ, LaRocco M, Kennedy V, Gentry LO. Effectiveness of 0.12% chlorhexidine gluconate oral rinse in reducing prevalence of nosocomial pneumonia in patients undergoing heart surgery. Am J Crit Care. 2002;11(6):567-70. PMID: 12425407.

4. World Health Organization. Hand Hygiene: Why, How & When? 2009. Available:http://www.who.int/gpsc/5may/Hand_Hygiene_Why_How_and_When_Brochure.pdf.
