# Supplementary material for: A simplified prevention bundle with dual hand hygiene audit reduces early-onset ventilator-associated pneumonia in cardiovascular surgery units: An interrupted time-series analysis
Source: PLoS One. 2017 Aug 2;12(8):e0182252. doi: 10.1371/journal.pone.0182252 (PMC5540591; doi:10.1371/journal.pone.0182252)
Supplement: S3 Appendix — (DOCX) [file pone.0182252.s003.docx]

**S3 Appendix**

**ARIMA analysis for monthly total VAP incidence**

| **Table A. The main statistics of different ARIMA models for monthly total VAP incidence** | | | | | | | | | | | | | | | |  |
| --- | --- | --- | --- | --- | --- | --- | --- | --- | --- | --- | --- | --- | --- | --- | --- | --- |
|  | | Model fit statistics | | | | | | | | Ljung-Box Q | | | | | | |
| Model | | Stationary R-squared | R-square | | MaxAPE | | | Normalized BIC | | Statistics | | DF | | Significance | | |
| ARIMA (0,0,0) | | .454 | .454 | | 224.482 | | | 5.963 | | 12.348 | | 18 | | .829 | | |
| ARIMA (1,0,0) | | .457 | .457 | | 222.485 | | | 6.091 | | 12.584 | | 17 | | .764 | | |
| ARIMA (0,1,0) | | .028 | -.326 | | 192.968 | | | 6.695 | | 18.535 | | 18 | | .421 | | |
| ARIMA (0,0,1) | | .457 | .457 | | 219.790 | | | 6.090 | | 12.732 | | 17 | | .754 | | |
| ARIMA (0,1,1) | | .411 | .196 | | 335.415 | | | 6.330 | | 12.603 | | 17 | | .762 | | |
| ARIMA (1,1,0) | | .266 | .000 | | 269.254 | | | 6.549 | | 9.844 | | 17 | | .910 | | |
| **ARIMA (1,0,1)** | | **.501** | **.501** | | **169.310** | | | **6.139** | | **15.535** | | **16** | | **.486** | | |
| ARIMA (1,0,2) | | .459 | .459 | | 194.887 | | | 6.220 | | 14.027 | | 16 | | .597 | | |
| ARIMA (1,0,3) | | .486 | .486 | | 119.005 | | | 6.169 | | 14.361 | | 16 | | .572 | | |
| ARIMA (1,0,4) | | .459 | .459 | | 227.796 | | | 6.219 | | 10.040 | | 16 | | .865 | | |
| ARIMA (2,0,0) | | .456 | .456 | | 209.524 | | | 6.092 | | 12.884 | | 17 | | .744 | | |
| ARIMA (2,0,1) | | .459 | .459 | | 193.181 | | | 6.219 | | 14.019 | | 16 | | .597 | | |
| ARIMA (2,0,2) | | .458 | .458 | | 203.197 | | | 6.222 | | 12.728 | | 16 | | .692 | | |
| ARIMA (2,0,3) | | .486 | .486 | | 90.341 | | | 6.170 | | 15.728 | | 16 | | .472 | | |
| ARIMA (2,0,4) | | .459 | .459 | | 222.656 | | | 6.220 | | 10.140 | | 16 | | .859 | | |
| ARIMA (3,0,0) | | .468 | .468 | | 169.292 | | | 6.070 | | 13.091 | | 17 | | .730 | | |
| ARIMA (3,0,1) | | .472 | .472 | | 153.681 | | | 6.196 | | 14.076 | | 16 | | .593 | | |
| ARIMA (3,0,2) | | .469 | .469 | | 153.108 | | | 6.201 | | 14.212 | | 16 | | .583 | | |
| ARIMA (3,0,3) | | .499 | .499 | | 157.871 | | | 6.142 | | 12.386 | | 16 | | .717 | | |
| ARIMA (3,0,4) | | .468 | .468 | | 183.674 | | | 6.203 | | 11.289 | | 16 | | .791 | | |
| ARIMA (3,0,5) | | .474 | .474 | | 172.155 | | | 6.192 | | 11.765 | | 16 | | .760 | | |
| MaxAPE, maximum absolute percentage error; BIC, Bayesian information criteria; DF, degree of freedom | | | | | | | | | | | | | | | |  |
| **Table B. ARIMA (1,0,1) model parameters** | | | | | | | | | | | | | | | |  |
|  | | | | | | | | Estimate | | Standard error | | T | | Significance | |  |
| Monthly total VAP incidence | | No transformation | | | Constant | | | -13.725 | | 47.227 | | -.291 | | .773 | |  |
|  |  |  |  |  | AR | | Lag 1 | .711 | | .289 | | 2.459 | | .020 | |  |
|  |  |  |  |  | MA | | Lag 1 | .995 | | 3.895 | | .255 | | .800 | |  |
| LnMVdays | | No transformation | | | Numerator | | Lag 0 | 32.242 | | 28.690 | | 1.124 | | .270 | |  |
| Phas2 | | No transformation | | | Numerator | | Lag 0 | 1.045 | | 10.198 | | .102 | | .919 | |  |
| Phas3 | | No transformation | | | Numerator | | Lag 0 | -26.417 | | 3.987 | | -6.627 | | .000 | |  |
| Phas4 | | No transformation | | | Numerator | | Lag 0 | -25.611 | | 6.529 | | -3.923 | | .000 | |  |
| LnMVdays, natural log of mechanical ventilator days.  The ARIMA model included monthly total VAP incidence as dependent variable as well as LnMVdays, phase (dummy phase 2 - 4) as independent variables. | | | | | | | | | | | | | | | |  |

**Fig A. The plots of residual autocorrelation function (ACF) and residual partial autocorrelation function (PACF) with ARIMA (1,0,1).**


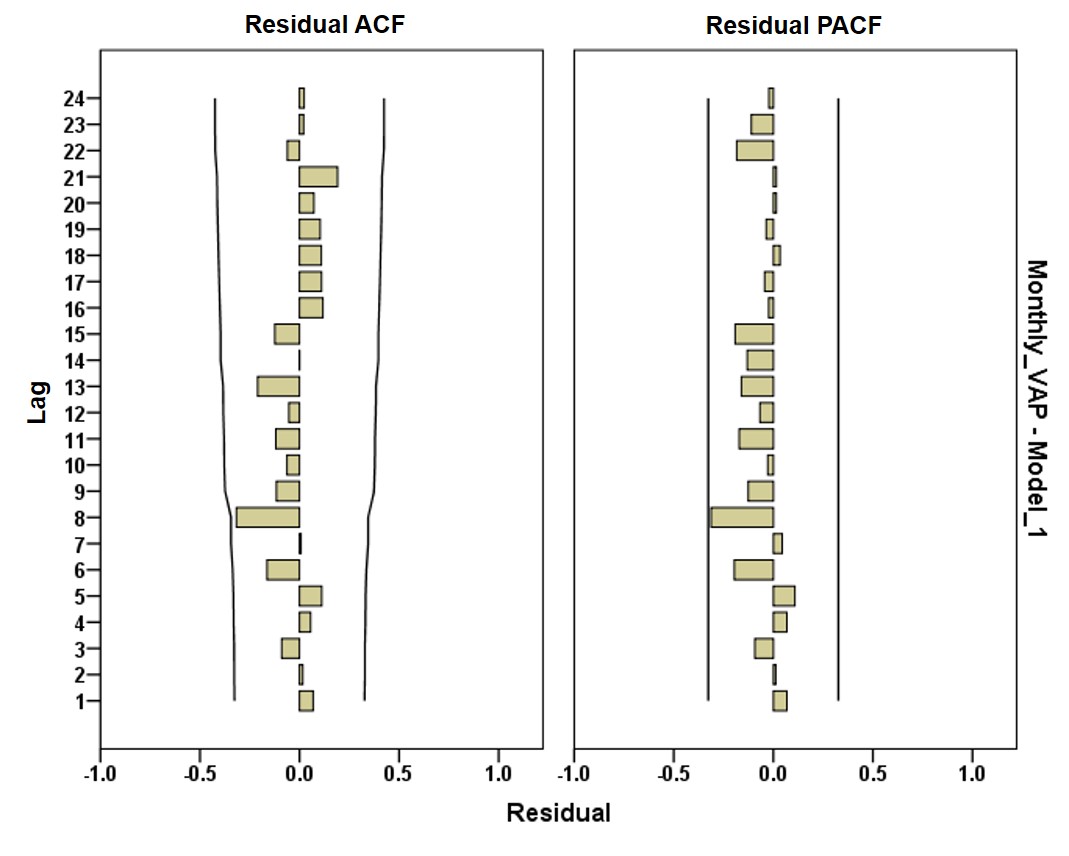


**Fig B. The time series plot of monthly total VAP incidence with ARIMA (1,0,1).**

(UCL, 95% upper conference limit; LCL, 95% lower conference limit)


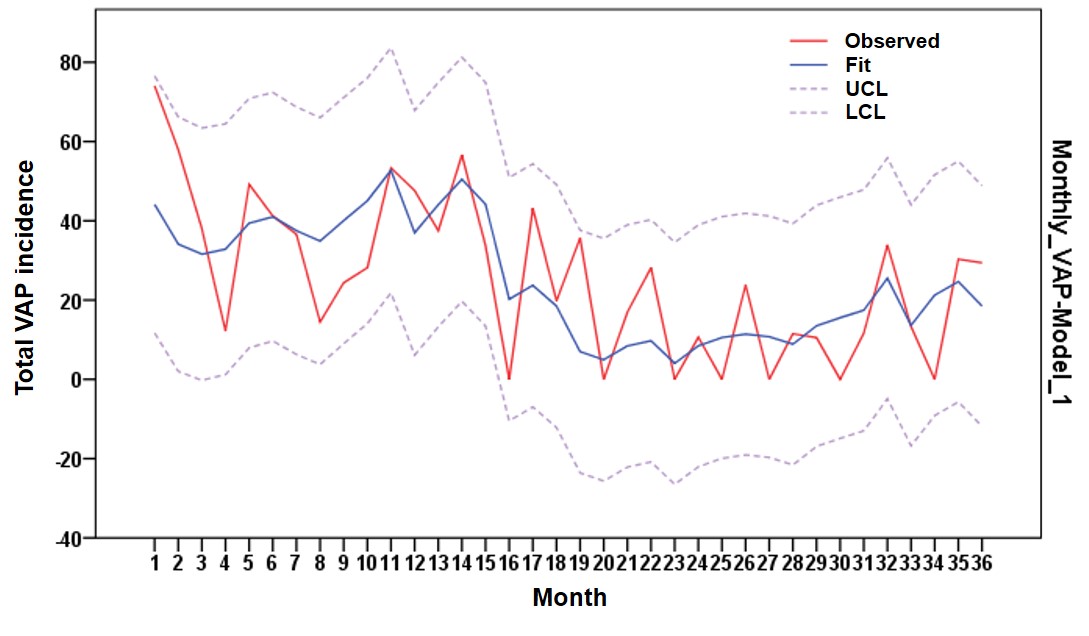


**Model summary:**

1. We adopted the ARIMA (1,0,1) as the best fit model to represent the serial change of monthly total VAP incidence (Table A). With this model, the R^2^ was 0.501, and the Ljung-Box Q statistic indicated the absence of statistically significant autocorrelations in residuals (Q = 15.535, df = 16, significance = 0.486). That was, the VAP incidence presented as a stationary time series data (Fig A). The monthly VAP incidence was independent, not been interfered with autocorrelation (Table A). The VAP bundle had an effect significantly greater than the underlying secular trend. The results of Poisson regression were comparable with those of ITS analysis.

2. In this model, the VAP incidence in phase 3 and 4 was significantly reduced in comparison with that in phase 1 (Table B).
