# Supplementary material for: A simplified prevention bundle with dual hand hygiene audit reduces early-onset ventilator-associated pneumonia in cardiovascular surgery units: An interrupted time-series analysis
Source: PLoS One. 2017 Aug 2;12(8):e0182252. doi: 10.1371/journal.pone.0182252 (PMC5540591; doi:10.1371/journal.pone.0182252)
Supplement: S1 Table — (DOCX) [file pone.0182252.s004.docx]

| **S1 Table. Microbiological spectrum in tracheal aspirates during different study phases.** | | | | | |
| --- | --- | --- | --- | --- | --- |
| **Microbiological Spectrum** | **Phase 1 (n=158)** | **Phase 2 (n=37)** | **Phase 3 (n=191)** | **Phase 4 (n=67)** | **Total (n=453)** |
| **No data** | 37 (23.4) | 5 (13.5) | 39 (20.4) | 14 (20.9) | 95 (21.0) |
| **No growth** | 43 (27.2) | 12 (32.4) | 70 (36.6) | 27 (40.3) | 152 (33.6) |
| **Gram-positive** |  |  |  |  |  |
| **Total** | 5 (3.2) | 1 (2.7) | 6 (3.1) | 1 (1.5) | 13 (2.9) |
| ***Staphylococcus aureus*** | 5 (3.2) | 1 (2.7) | 5 (2.6) | 1 (1.5) | 12 (2.6) |
| ***Streptococcus* spp.** | 0 | 0 | 1 (0.5) | 0 | 1 (0.2) |
| **Gram-negative** |  |  |  |  |  |
| **Total** | 65 (41.1) | 15 (40.5) | 64 (33.5) | 22 (32.8) | 166 (36.6) |
| ***Pseudomonas aeruginosa*** | 16 (10.1) | 4 (10.8) | 10 (5.2) | 3 (4.5) | 33 (7.3) |
| ***Acinetobacter baumannii*** | 12 (7.6) | 3 (8.1) | 24 (12.6) | 4 (6.0) | 43 (9.5) |
| ***Escherichia coli*** | 4 (2.5) | 0 | 0 | 4 (6.0) | 8 (1.8%) |
| ***Klebsiella pneumonia*** | 14 (8.9) | 6 (16.2)^a^ | 10 (5.2)^a^ | 4 (6.0) | 34 (7.5) |
| ***Enterobacter* spp*.*** | 6 (3.8) | 2 (5.4) | 9 (4.7) | 3 (4.5) | 20 (4.4) |
| **Other GNB** | 13 (8.2) | 0 | 11 (5.8) | 4 (6.0) | 28 (6.2) |
| **Fungi** |  |  |  |  |  |
| **Total** | 8 (5.1) | 4 (10.8) | 12 (6.3) | 3 (4.5) | 27 (6.0) |
| ***Candida* spp.** | 8 (5.1) | 4 (10.8) | 11 (5.8) | 3 (4.5) | 26 (5.7) |
| ***Aspergillus* spp.** | 0 | 0 | 1 (0.5) | 0 | 1 (0.2) |
| **Drug resistant bacteria** |  |  |  |  |  |
| **Total** | 16 (10.0) | 6 (16.2) | 16 (8.4) | 3 (4.5) | 41 (9.1) |
| **MRSA** | 5 (3.2) | 1 (2.7) | 3 (1.6) | 0 | 9 (2.0) |
| **CRAB** | 8 (5.1) | 2 (5.4) | 13 (6.8) | 1 (1.5) | 24 (5.3) |
| **ESBL(+) *E.Coli* & *K.P.*** | 3 (1.9) | 3 (8.1) | 0 | 2 (3.0) | 8 (1.8) |
| GNB, gram negative bacilli; MRSA, Methicillin-resistant *Staphylococcus aureus*; *E. coli*, *Escherichia coli; K.P.,* *Klebsiella pneumonia;* CRAB, carbapenem-resistant *Acinetobacter baumannii;* ESBL(+), extended spectrum beta-lactamase producing bacilli.  Categorical variables are presented as counts and percentages in parentheses.  ^a^Chi-square test, *P* = 0.017, phase 2 *vs.* 3. All *P* value > 0.05, except for the incidence of *Klebsiella pneumonia.* | | | | | |
