# Supplementary material for: A simplified prevention bundle with dual hand hygiene audit reduces early-onset ventilator-associated pneumonia in cardiovascular surgery units: An interrupted time-series analysis
Source: PLoS One. 2017 Aug 2;12(8):e0182252. doi: 10.1371/journal.pone.0182252 (PMC5540591; doi:10.1371/journal.pone.0182252)

**S1 Fig. The modified clinical pulmonary infection score categorized by the presence or absence of ventilator-associated pneumonia in total patients or in patients of different study phases.**

Data are presented with mean SD (error bar). * *P* < 0.05, t-test, *vs.* VAP, respectively.


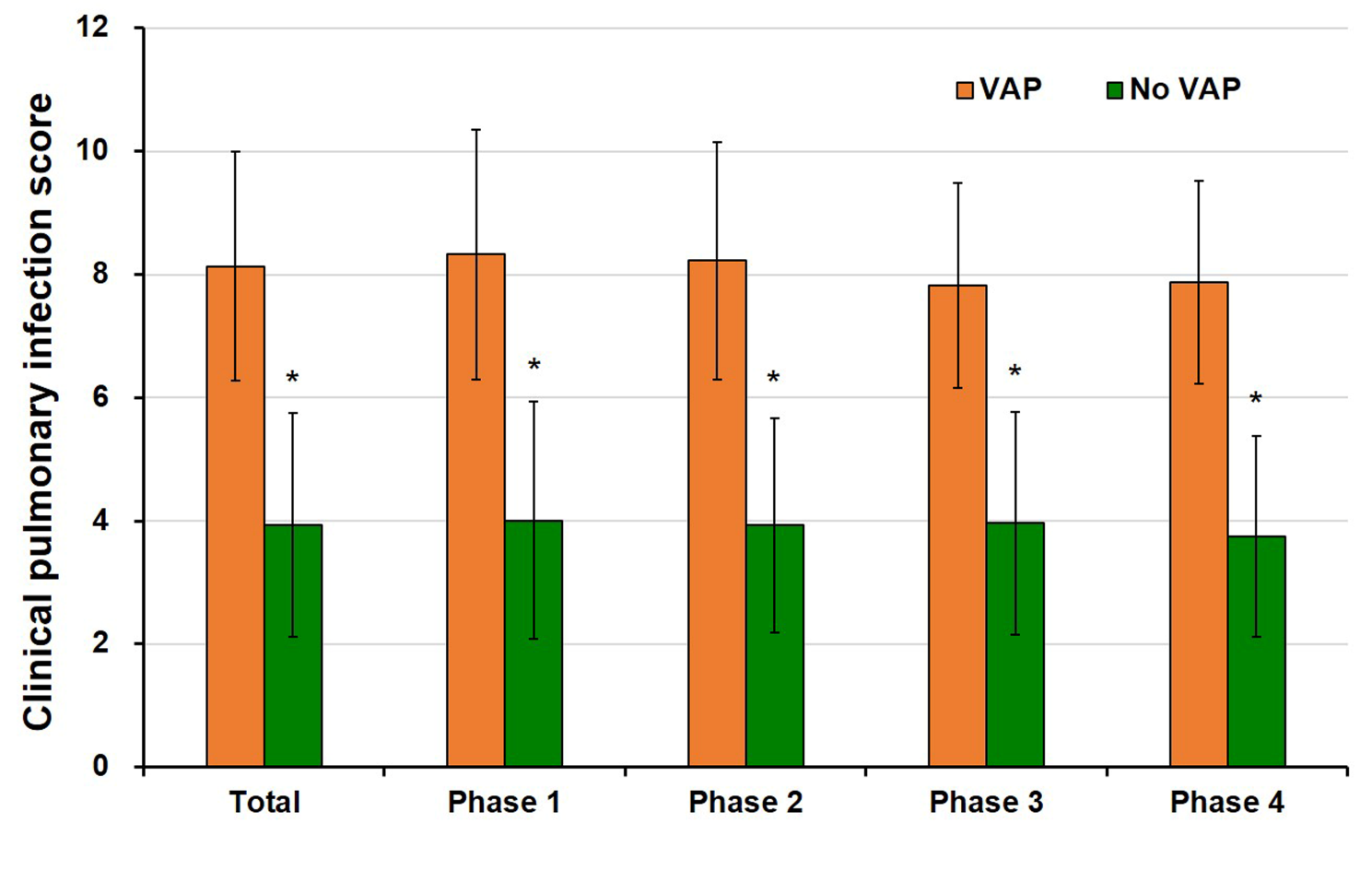

Supplement: S1 Fig — (DOCX) [file pone.0182252.s005.docx]
