# Supplementary material for: A simplified prevention bundle with dual hand hygiene audit reduces early-onset ventilator-associated pneumonia in cardiovascular surgery units: An interrupted time-series analysis
Source: PLoS One. 2017 Aug 2;12(8):e0182252. doi: 10.1371/journal.pone.0182252 (PMC5540591; doi:10.1371/journal.pone.0182252)

**S2 Fig. Changes of compliance for unaware external hand hygiene audit (eHH) in different healthcare workers during different phases.** *Chi-Square test, *P* < 0.05.


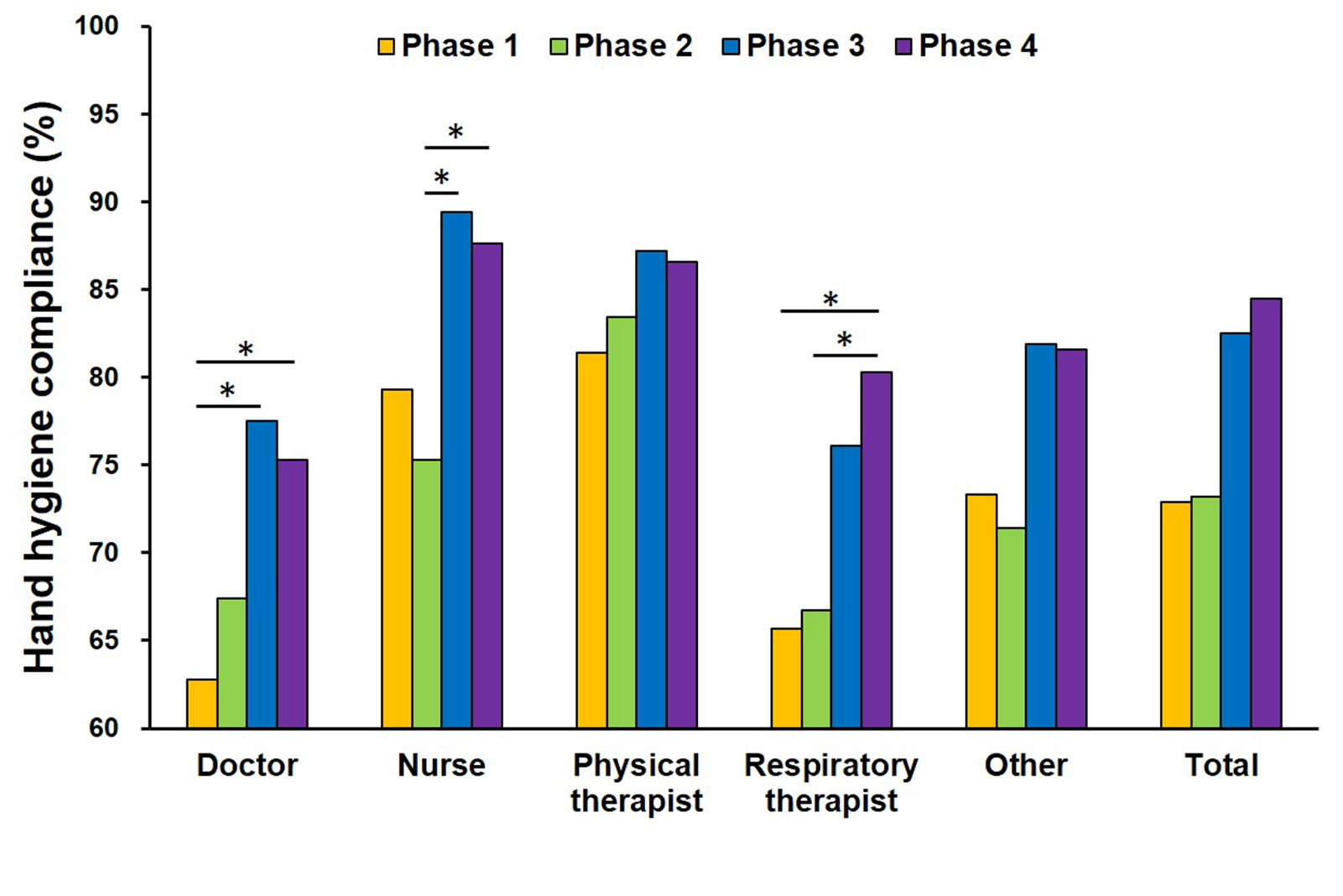

Supplement: S2 Fig — (DOCX) [file pone.0182252.s006.docx]
